# Late Replicating Domains Are Highly Recombining in Females but Have Low Male Recombination Rates: Implications for Isochore Evolution

**DOI:** 10.1371/journal.pone.0024480

**Published:** 2011-09-20

**Authors:** Catherine J. Pink, Laurence D. Hurst

**Affiliations:** Department of Biology and Biochemistry, University of Bath, Bath, United Kingdom; Virginia Tech Virginia, United States of America

## Abstract

In mammals sequences that are either late replicating or highly recombining have high rates of evolution at putatively neutral sites. As early replicating domains and highly recombining domains both tend to be GC rich we *a priori* expect these two variables to covary. If so, the relative contribution of either of these variables to the local neutral substitution rate might have been wrongly estimated owing to covariance with the other. Against our expectations, we find that sex-averaged recombination rates show little or no correlation with replication timing, suggesting that they are independent determinants of substitution rates. However, this result masks significant sex-specific complexity: late replicating domains tend to have high recombination rates in females but low recombination rates in males. That these trends are antagonistic explains why sex-averaged recombination is not correlated with replication timing. This unexpected result has several important implications. First, although both male and female recombination rates covary significantly with intronic substitution rates, the magnitude of this correlation is moderately underestimated for male recombination and slightly overestimated for female recombination, owing to covariance with replicating timing. Second, the result could explain why male recombination is strongly correlated with GC content but female recombination is not. If to explain the correlation between GC content and replication timing we suppose that late replication forces reduced GC content, then GC promotion by biased gene conversion during female recombination is partly countered by the antagonistic effect of later replicating sequence tending increase AT content. Indeed, the strength of the correlation between female recombination rate and local GC content is more than doubled by control for replication timing. Our results underpin the need to consider sex-specific recombination rates and potential covariates in analysis of GC content and rates of evolution.

## Introduction

In mammals autosomal regions differ in the rate of evolution of putatively neutral sites [1], [2]. As all autosomes replicate the same number of times in any given germline, this heterogeneity can not be accounted for in terms of the number of cell divisions, this variable being thought to be important in explaining, in part, why X, Y and autosome evolve at different rates [3], [4], [5], [6], [7]. Two important variables have been conjectured to be important in explaining the intra-autosome heterogeneity. Recently several reports have supported the possibility that genomic domains have characteristic replication times through the cell cycle, that these timings are evolutionarily conserved and that early replicating sequence, for reasons unknown, have low neutral rates of evolution [8], [9], [10]. Comparably, genomic domains have characteristic and conserved (on the megabase scale) recombination rates, with high rates being associated with high rates of evolution at putatively neutral sites [11], [12], [13], [14], [15]. Again, the underlying cause is unclear but this might reflect a mutagenic effect of recombination [16], [17], [18] or the action of biased gene conversion. Due to biases in the mismatch repair process [19], the latter process tends to favour fixation of G/C over A/T and has thus been suggested as a mechanism for the origin or maintenance of isochores ([20] and references therein, [21]) and can increase rates of evolution that are not at equilibrium [22].

What has yet to be established is the extent to which these two variables, replication timing and recombination rate, are independent predictors of neutral rates of evolution. *A priori* we might suppose that a fuller appreciation of the role of both of these would need both parameters to be considered synchronously. Domains of high recombination have high GC content (possibly owing to the action of biased gene conversion). Similarly, early replicating domains tend to be GC rich [8], [23], [24]. Thus we might expect a) early replicating domains to be high recombination zones and b) as early replication is associated with low neutral substitution rates and high recombination is associated with high rates, that the two effects mask each other leading to an underestimate of the effect each has when either is considered in isolation. Here then we investigate this issue both at the genic level and also with regard to the enigmatic between-autosome variation in neutral rates [11], [25], [26].

An increasing body of evidence suggests that the effect of recombination on weak-to-strong (A/T to G/C) substitutions correlates more strongly with rates in males than in females [13], [27], [28], [29], [30], [31]. The reasons why this might be have not yet been elucidated, although a mechanistic difference in meiotic recombination has been suggested [31]. Given the potential importance of sex-specific recombination rates we consider not just sex-averaged recombination rates but repeat all the analyses using both male- and female-specific recombination rates.

With the inclusion of sex-specific recombination rates our analysis differs from that of Chen *et al.* [10], who argue that the effect of replication timing on neutral evolutionary rates is not explained by recombination. This group, however, only examined sex-averaged rates. As we show here, such an analysis misses the more complex effects of sex-specific recombination rates. Our analysis is also different to that of Clément and Arndt [32] who noticed that GC content in rodents is well predicted by male specific recombination rates but not by female specific ones, and thus chose to ignore further consideration of female recombination as a potentially important cause of GC content. We recover the same “raw” results but show that the effect of female recombination on GC content is majorly underestimated if one fails to allow for covariance with replication timing.

## Methods

### Estimating intronic substitution rates

Intronic substitution rates were calculated using the same methodology as for Pink and Hurst (for details see [8]). Briefly, orthologous mouse and rat genes were originally defined by MGI [33] and further filtered to ensure similarity of exon number and phase. Introns were aligned individually using Lagan [34] prior to removal of first introns and 30 bp at intron ends, both thought to be under selective constraints [35]. The data set was further purged of introns containing more runs of conserved bases than would be expected by chance (see [11] for details). Remaining introns were concatenated by gene before calculation of intronic substitution rates (*K*_i_), with correction for multiple hits according to Tamura and Kumar [36].

### Estimating GC content

Mouse GC content was calculated directly from genomic sequences at intronic sites using repeat-masked sequences to control for the possible influence of AT rich transposable element insertions. Genomic sequence files for the mouse genome mm9 (NCBI build 37, July 2007) were obtained from the UCSC table browser located at http://genome.ucsc.edu/ [37]. Dubious RefSeqs that either were present in more than one copy, were found to be located on random or multiple chromosomes, that were not located on a single strand, or that were intronless were identified and removed from the analysis. Intronic sequences pertaining to RefSeqs where exons contained premature stop codons or incomplete codons and that did not begin and end with correct start and termination codons were identified and purged from the analysis. For each intron 30 bp were removed from both ends to control for the possible influence of conserved splice sites [38]. First introns were also removed, these known to be unusually slow evolving [35], [39]. Remaining intronic sequences were then concatenated by RefSeq. Counts of each base (A, T, C, G and N) were then made from which GC content (GC) was calculated as [(G+C)/(A+T+G+C)]. Repeat-masked and unmasked GC_i_ were, as expected, found to covary significantly (Spearman's ρ = 0.983, *P*<2.2×10^−16^; *n* = 18775, Figure S1).

### The rearrangement index

Each mouse autosome was assigned a rearrangement index, a measure of the probability that the rat orthologs of any two randomly selected genes on a given mouse autosome are not both located on the same rat autosome. For a focal mouse autosome, two genes were randomly sampled and the location of their rat orthologs determined. From 10,000 samplings, the number of occasions on which the rat orthologs were located on different chromosomes was counted (*n*). The index of rearrangement (RI) was then calculated for the autosome as (*n*/10,000), such that highly rearranged autosomes were assigned higher indices. Note that this rearrangement index does not quantify the extent of intra-chromosomal rearrangements such as inversions.

### Assaying replication time

Replication times in *Mus musculus* were determined by Hiratani *et al.* [40] who provide four replication timing datasets. Three were derived from separate embryonic stem cell lines (ECSs). Inclusion of a fourth dataset derived from induced pluripotent stem cells (iPS) had previously been justified and so was again included. These datasets were downloaded in files RD_TT2ESCave_Sm300_081128.txt, RD_iPSave_Sm300_081128.txt, RD_D3ESCave_Sm300_081128.txt and RD_46CESCave_Sm300_081128.txt from the ReplicationDomain website [41]. Array probe positions were converted from mouse build mm8 (NCBI build 36) to build mm9 (NCBI build 37) using the UCSC liftOver tool and associated chain file mm8ToMm9.over.chain. All probes located within the limits of the coding sequence of a RefSeq were then identified. Of the 21471 RefSeqs, 14881 were assigned sufficient replication times to be able to test for normality of distribution. Kolmogorov smirnov tests showed that replication times of 5126 RefSeqs (35.5% of those tested) were normally distributed while 9755 (65.6% of those tested) had skewed distributions. Median replication times were therefore assigned to each RefSeq. It should be noted that use of mean replication times did not qualitatively alter the findings (see Tables S1, S2 and S3).

### Methods to estimate the local recombination rate

In contrast to our prior analysis [11] that utilised recombination rates in rat, here we used recombination rates in mouse. This enabled comparison of the relative contributions of recombination and replication time to rates of evolution in a single species. The genetic map used was originally determined by Shifman *et al.* [42], derived from a large heterogeneous mouse population descended from eight inbred strains. Cox *et al.* [43], having identified two methodological problems with the Shifman genetic map, subsequently updated this dataset and incorporated SSLP markers from other genetic maps to generate a revised standard genetic map for the mouse. The map consists of 10,195 SNPs at an average density of 258 Kb (99% of SNP intervals <500 kb, 81.2% <250 kb) and is based on 3546 meioses. This revised genetic map was therefore used for this analysis. The genetic map was downloaded from http://cgd.jax.org/mousemapconverter/Revised_HSmap_SNPs.csv - Mouse Map Data (Base Pair to centimorgan mapping). SNP positions had already been updated to the current mouse build mm9 (NCBI build 37). In addition to the SNP ID, the chromosome and bp physical position of the SNP, this file contained three genetic maps: a male-specific map, a female-specific map and a sex-averaged map. Assignment of recombination rates to RefSeqs was performed using a number of alternative methodologies:

1. Chromosomal recombination rates are generally calculated from the most proximal and distant markers. Doing so captures all recombination events along the chromosome. Application of a similar methodology to individual RefSeqs involved identification of the two flanking SNPs. The physical and genetic positions of these markers could then be used to calculate the recombination rate of the intervening region in which the RefSeq was located. The median distance between the edge of a gene and the flanking marker was 155346.5 bp.
2. Human recombination rates, such as the deCODE, Marshfield and Genethon genetic maps, are available as additional tracks on the UCSC genome browser. These are essentially weighted averages, whereby the recombination rate between immediately flanking markers is calculated and, assuming a linear genetic distance between markers, each base within the interval is assigned the recombination rate. 1 Mb windows are then assigned recombination rates based on the average rate of the bases contained within the window. A similar method was therefore applied to genes, albeit without smoothing over 1 Mb windows. RefSeqs were assigned mean recombination rates weighted by the base pair overlap of the marker interval with the gene. This was, in effect, the same as assigning each base pair within the gene a recombination rate and then taking a mean across all base pairs. A ‘weighted median’ was also calculated by assigning each base pair within the gene a recombination rate and then taking a median across all base pairs, since the per-base pair recombination rates of over 1000 genes had skewed distributions.
3. A method similar to that applied to the assignment of replication times to each RefSeq was also used. Here, for each chromosome the recombination rate between every neighbouring pair of SNPs was calculated. Each SNP interval that overlapped with a given RefSeq was identified and the average mean and median recombination rate of these intervals was taken. Note that for genes that lacked internal SNPs, this resulted in the same genic recombination rate as for method 1.
4. To reduce noise, smoothing techniques were also applied. Two methods of smoothing were used and in each case, both means and medians were used, thus giving four smoothed rates. Firstly, all markers within a 2 Mb window of the flanking interval were identified (1 Mb in each direction from the 5′ SNP). Recombination rates between each pair of markers were calculated, again assuming a linear genetic distance between markers. The average recombination rate of all these marker intervals was taken and assigned to the focal interval (denoted average-smoothed^1^ in the text). Secondly, in addition to the focal interval, these 2 Mb averaged recombination rates were assigned to every interval within the 2 Mb window. Once this process had been repeated using all intervals as a focal point for the 2 Mb smoothing, the average of all smoothed rates assigned to a window was taken (denoted average-smoothed^2^ in the text). Finally, these four smoothed rates were assigned to genes using the same technique as described in method 3.

**Figure 1:**
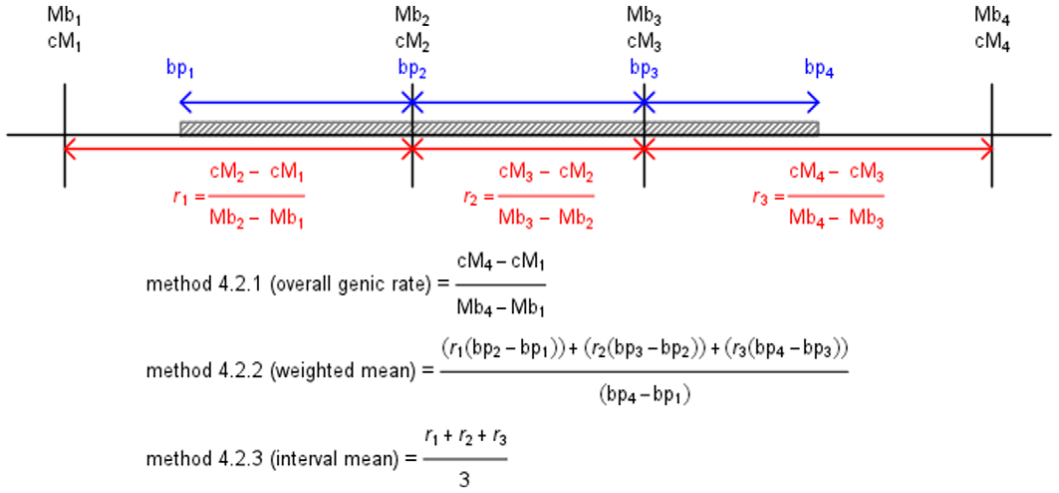
Methodologies used to generate gene-focused recombination rates. Representation of the methods used to calculate gene-focused recombination rates (methods 4.2.1, 4.2.2 and 4.2.3). Note that this diagram is for descriptive purposes only and is not to scale. For simplicity, only calculations for mean rates are shown. The grey region is a gene. Vertical black lines represent four SNP markers with physical (Mb) and genetic (cM) positions. Blue arrows represent the base pairs of the gene overlapping with each intervening SNP interval. In red are recombination rates (*r_x_*) between pairs of neighbouring markers.

**Figure 2:**
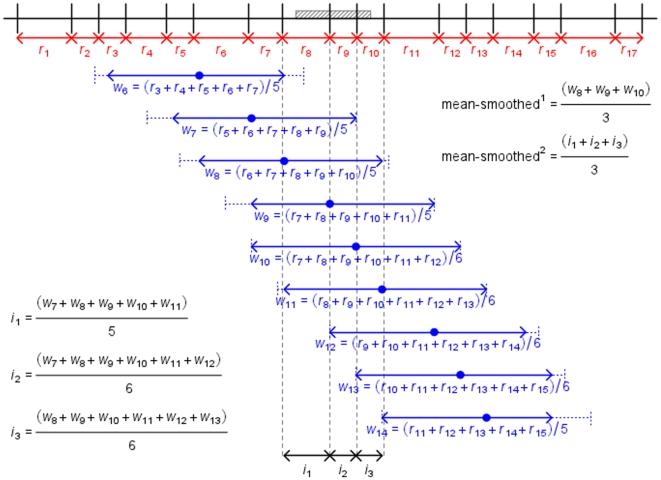
Methodologies used to generate smoothed recombination rates. Representation of methods used to calculate smoothed recombination rates (method 4.2.4). Note that this diagram is for descriptive purposes only and is not to scale. For simplicity, only calculations for mean rates are shown. The grey region is a gene. Vertical black lines are SNP markers. In red are recombination rates between pairs of neighbouring markers (*r_x_*). Dashed blue lines represent 1 Mb windows either side of a focal SNP. Solid blue arrows represent all intervals within this window, over which recombination rates are averaged (*w_x_*, averaged-smoothed^1^). For three intervals, averages of all window averages covering the interval are shown (i*_x_*, average-smoothed^2^).

For visual explanation of these methods see Figure 1 (genic) and Figure 2 (smoothed). These alternative methodologies were applied to both the sex-averaged, male-specific and female-specific data (for examples see Figures S2 and S3). Every statistical analysis that included recombination rate as a parameter was repeated using every method described.

### Data set dimensions

For the analyses presented here, the final dataset was purged of all sex-linked RefSeqs. In addition, only RefSeqs that had been assigned data for all variables of interest - intronic substitution rates (*K*_i_); GC content (GC); timing of replication (RT); and recombination rate (RR) - were retained, thus ensuring that the sample size, and therefore statistical power, was comparable across all analyses. The resulting dataset comprised 3549 genes.

For all genic datasets, Kolmogorov Smirnov tests were applied, showing that data were skewed and could not be normalised. Similarly, Kolmogorov Smirnov tests performed on data assigned to individual autosomes showed that all data types were also skewed. As such, for analyses of between-autosomal variation, the median autosomal value for each data type was taken. To these autosomal medians, the overall recombination rate between the most proximal and distal markers on the chromosome, plus the rearrangement indices were added. Finally, for each data type the distributions of the 19 autosomal values were found to be normally distributed, thus enabling the use of parametric tests for analyses at the autosomal level.

### Calculation of partial spearman correlations

Partial Spearman's correlations between *x* and *y*, controlling for *z* (ρ_xy.z_), were calculated as follows:
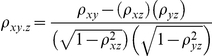
where ρ_xy_ are Spearman's correlations between the two variables indicated by the subscript. Significance was determined by randomly reassigning *y* to each gene, without replacement, and then re-calculating the partial Spearman's correlation (ρ_xy.z_). This process was repeated 1000 times and the number of occasions (*n*) on which the strength of the randomised ρ_xy.z_ exceeded that of the original, was used to calculate *P* as *P* = (*n*+1)/(1000+1).

## Results

### A sex-specific relationship between replication time and recombination rate at the genic level

We start by asking two sets of questions. First, is it robustly found that replication time and the local recombination rate, defined multiple ways, both correlate with the intronic substitution rate? Second, is it true that recombination and replication time covary as we presume? If the second is true then the former results would need to be analysed under a covariate controlled model.

Regarding the first issue, the previously observed [8] relationship between replication timing and rates of intronic evolution was confirmed in the new dataset (Spearman's ρ = −0.081, *P* = 1.35×10^−6^). Note that because of how the replication timing data was structured, an increase in any parameter as S-phase proceeds yields a negative correlation and *vice versa*. The relationship between recombination rates and intronic substitution rates was more complex, being sensitive to both gender and methodology. In general, all recombination rate datasets that involved an element of smoothing resulted in stronger correlations with *K*_i_ than the gene-focused curation methods such as overall rates, weighted, base pair and interval averages (Table 1). For smoothed rates, the magnitude of the relationship was similar to that observed for replication times (for mean-smoothed^2^ sex-averaged recombination rates Spearman's ρ = 0.1, *P* = 2.39×10^−9^) whereas for unsmoothed rates, the strength of the relationship was approximately half that for replication times (for overall sex-averaged recombination rates Spearman's ρ = 0.045, *P* = 0.0073).

**Table 1:** Spearman's correlations using alternative recombination rate datasets.

Variable			Statistic	Overall genic	Weighted mean	Base pair median	Interval mean	Interval median	Mean smoothed^1^	Median smoothed^1^	Mean smoothed^2^	Median smoothed^2^
X	Y	Z										
*K*_i_	GC	RR_SA_	ρ	−0.081	−0.081	−0.081	−0.081	−0.081	−0.089	−0.083	−0.092	−0.081
			*P*	0.001	0.001	0.001	0.001	0.001	0.001	0.001	0.001	0.001
		RR_M_	ρ	−0.08	−0.079	−0.079	−0.079	−0.079	−0.085	−0.079	−0.088	−0.077
			*P*	0.001	0.001	0.001	0.001	0.001	0.001	0.001	0.001	0.001
		RR_F_	ρ	−0.08	−0.08	−0.08	−0.08	−0.08	−0.081	−0.079	−0.083	−0.078
			*P*	0.001	0.001	0.001	0.001	0.001	0.001	0.001	0.001	0.001
	RT	RR_SA_	ρ	−0.08	−0.08	−0.081	−0.08	−0.08	−0.086	−0.079	−0.087	−0.077
			*P*	0.001	0.001	0.001	0.001	0.001	0.001	0.001	0.001	0.001
		RR_M_	ρ	−0.081	−0.081	−0.081	−0.081	−0.081	−0.089	−0.082	−0.09	−0.082
			*P*	0.001	0.001	0.001	0.001	0.001	0.001	0.001	0.001	0.001
		RR_F_	ρ	−0.078	−0.078	−0.079	−0.078	−0.079	−0.076	−0.078	−0.076	−0.077
			*P*	0.001	0.001	0.001	0.001	0.001	0.001	0.001	0.001	0.001
	RR_SA_	-	ρ	0.045	0.041	0.038	0.045	0.043	0.095	0.084	0.1	0.088
			*P*	0.007	0.015	0.023	0.007	0.01	1.24×10^−8^	5.14×10^−7^	2.39×10^−9^	1.67×10^−7^
	RR_M_	-	ρ	0.015	0.01	0.006	0.013	0.009	0.057	0.054	0.058	0.057
			*P*	0.379	0.544	0.73	0.454	0.574	0.001	0.001	0.001	0.001
	RR_F_	-	ρ	0.044	0.041	0.039	0.044	0.043	0.084	0.071	0.092	0.08
			*P*	0.009	0.015	0.02	0.008	0.01	5.22×10^−7^	2.62×10^−5^	4.25×10^−8^	1.81×10^−6^
	RR_SA_	GC	ρ	0.051	0.047	0.044	0.05	0.049	0.104	0.088	0.111	0.09
			*P*	0.002	0.003	0.003	0.001	0.005	0.001	0.001	0.001	0.001
	RR_M_	GC	ρ	0.021	0.016	0.013	0.019	0.016	0.067	0.055	0.07	0.056
			*P*	0.114	0.178	0.234	0.137	0.157	0.001	0.003	0.001	0.001
	RR_F_	GC	ρ	0.047	0.044	0.043	0.047	0.046	0.087	0.071	0.096	0.08
			*P*	0.004	0.005	0.005	0.002	0.004	0.001	0.001	0.001	0.001
	RR_SA_	RT	ρ	0.042	0.039	0.037	0.043	0.041	0.1	0.082	0.105	0.084
			*P*	0.008	0.01	0.012	0.005	0.014	0.001	0.001	0.001	0.001
	RR_M_	RT	ρ	0.017	0.012	0.01	0.015	0.012	0.068	0.055	0.07	0.058
			*P*	0.162	0.209	0.3	0.197	0.213	0.001	0.001	0.001	0.001
	RR_F_	RT	ρ	0.038	0.035	0.035	0.039	0.038	0.08	0.067	0.088	0.076
			*P*	0.013	0.015	0.022	0.013	0.015	0.001	0.001	0.001	0.001
GC	RT	RR_SA_	ρ	0.296	0.295	0.294	0.295	0.295	0.29	0.294	0.289	0.294
			*P*	0.001	0.001	0.001	0.001	0.001	0.001	0.001	0.001	0.001
		RR_M_	ρ	0.292	0.291	0.29	0.292	0.291	0.283	0.293	0.278	0.293
			*P*	0.001	0.001	0.001	0.001	0.001	0.001	0.001	0.001	0.001
		RR_F_	ρ	0.296	0.296	0.295	0.296	0.296	0.295	0.293	0.296	0.293
			*P*	0.001	0.001	0.001	0.001	0.001	0.001	0.001	0.001	0.001
	RR_SA_	-	ρ	0.067	0.068	0.077	0.067	0.07	0.102	0.048	0.126	0.031
			*P*	6.44×10^−5^	4.80×10^−5^	4.51×10^−6^	6.46×10^−5^	2.76×10^−5^	1.07×10^−9^	0.004	4.45×10^−14^	0.064
	RR_M_	-	ρ	0.078	0.078	0.085	0.078	0.081	0.111	0.01	0.144	−0.016
			*P*	3.33×10^−6^	3.19×10^−6^	3.85×10^−7^	3.79×10^−6^	1.39×10^−6^	3.39×10^−11^	0.539	0	0.343
	RR_F_	-	ρ	0.038	0.037	0.048	0.035	0.041	0.027	0.005	0.044	−0.007
			*P*	0.025	0.026	0.005	0.036	0.015	0.104	0.753	0.008	0.692
	RR_SA_	RT	ρ	0.081	0.08	0.083	0.079	0.08	0.092	0.057	0.116	0.046
			*P*	0.001	0.001	0.001	0.001	0.001	0.001	0.001	0.001	0.005
	RR_M_	RT	ρ	0.074	0.073	0.074	0.073	0.074	0.079	0.008	0.109	−0.02
			*P*	0.001	0.001	0.001	0.001	0.001	0.001	0.318	0.001	0.116
	RR_F_	RT	ρ	0.063	0.061	0.064	0.059	0.061	0.048	0.021	0.064	0.011
			*P*	0.001	0.001	0.001	0.001	0.001	0.007	0.104	0.001	0.252
RT	RR_SA_	-	ρ	−0.034	−0.027	−0.009	−0.03	−0.022	0.048	−0.024	0.051	−0.045
			*P*	0.041	0.102	0.578	0.074	0.188	0.005	0.148	0.002	0.008
	RR_M_	-	ρ	0.025	0.028	0.049	0.026	0.034	0.122	0.01	0.138	0.01
			*P*	0.135	0.095	0.004	0.122	0.041	2.6×10^−13^	0.56	1.2×10^−16^	0.547
	RR_F_	-	ρ	−0.076	−0.07	−0.046	−0.073	−0.061	−0.062	−0.051	−0.056	−0.059
			*P*	6.34×10^−6^	3.19×10^−5^	0.006	1.37×10^−5^	0	0	0.003	0.001	0
	RR_SA_	GC	ρ	−0.056	−0.05	−0.033	−0.052	−0.045	0.019	−0.04	0.015	−0.056
			*P*	0.001	0.003	0.02	0.001	0.005	0.14	0.009	0.189	0.001
	RR_M_	GC	ρ	0.002	0.005	0.025	0.003	0.011	0.095	0.007	0.102	0.015
			*P*	0.443	0.376	0.067	0.423	0.255	0.001	0.349	0.001	0.171
	RR_F_	GC	ρ	−0.091	−0.084	−0.063	−0.087	−0.076	−0.073	−0.054	−0.072	−0.06
			*P*	0.001	0.001	0.001	0.001	0.001	0.001	0.001	0.001	0.001

Spearman's correlations at the genic level for each alternative method used to curate genic recombination rate data where: Z = the controlling variable used in partial Spearman's correlations between variables X and Y; *K*_i_ = intronic substitution rate between mouse and rat; RT = median replication time for each gene; GC = repeat-masked intronic G+C content for each gene; RR_SA_, RR_M_ and RR_F_ = sex-averaged, male and female genic recombination rates respectively.

Interestingly, the relationship between substitution rates and recombination appears to be driven by recombination in females: all female-specific recombination rates showing significant positive correlations with *K*_i_, whereas for male-specific recombination rates, correlation coefficients for smoothed datasets are approximately half the magnitude of those for females and for gene-focused datasets no significant relationships were observed (Table 1). This was surprising, as weak-to-strong substitutions associated with GC biased gene conversion (gBGC) in primates have been found to covary more strongly with male-specific recombination rates [13], [27], [28], [29], [30], [31].

**Figure 3:**
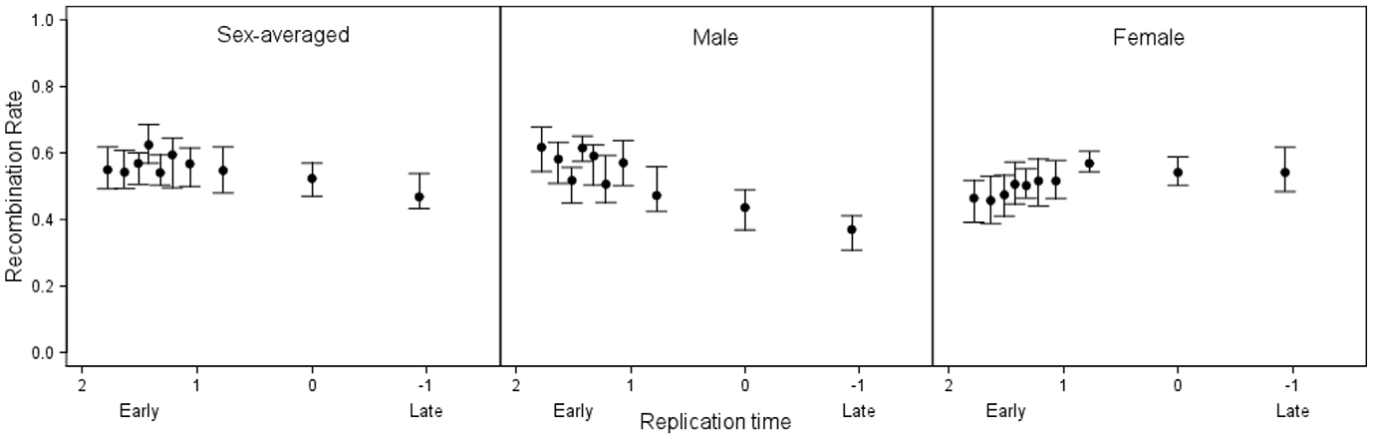
Sex-specific relationships between replication time and recombination rate. Relationships between replication time and sex-averaged, male-specific and female-specific recombination rates. Data shown are mean-smoothed^2^ data binned by median replication time where points are the median of each equally sized bin ± 95% confidence intervals.

As to the second issue, whether timing of replication and recombination rates covary, unexpectedly we found that no consistent relationship was observed for sex-averaged recombination rates, with both increasing and declining rates associated with sequences that replicate later during S-phase (Table 1). Closer examination suggests that this result reflects differences between males and females (Figure 3). Female recombination rates were consistently found to be higher in regions that replicate later during S-phase, irrespective of smoothing (for overall female recombination rates Spearman's ρ = −0.076, *P* = 6.34×10^−6^, Table 1). In contrast, genes that replicated later were found to have significantly lower male-specific recombination rates for some methodologies (e.g. for mean-smoothed^2^ male recombination rates Spearman's ρ = 0.138, *P* = 1.21×10^−16^) whereas for other measures no relationship was observed (e.g. for overall male recombination rates Spearman's ρ = 0.025, *P* = 0.135, Table 1).

### Weak interference between replication timing and sex specific recombination rates in determining intronic substitution rates

Given this result we need to ask whether the high substitution rate of late replicating sequence is due to it having high recombination rates in females and vice versa. Similarly, we can ask whether the impact of male recombination on rates of evolution have been underestimated as male-specific recombination rates are low where the effect of replication is also weakest.

We find that controlling for female recombination rates reduces the strength of the relationship between *K*_i_ and replication time. This is the case for all female-specific datasets (for the uncontrolled analysis Spearman's ρ = −0.081, *P* = 1.35×10^−6^; controlling for overall female recombination partial Spearman's ρ = −0.078, *P* = 0.001, Table 1), although the effect appears quite modest. Similarly, controlling for replication time reduces the strength of the relationship between intronic substitution rate and all measures of female-specific recombination rate (for the uncontrolled relationship between *K*_i_ and overall female recombination, Spearman's ρ = 0.044, *P* = 0.0090; controlling for replication time partial Spearman's ρ = 0.038, *P* = 0.013, Table 1).

In contrast, the higher male-specific recombination rates of early replicating sequences might mask the impact of replication time on rates of evolution and *vice versa*. When controlling for male recombination we might therefore expect the magnitude of the relationship between *K*_i_ and replication time to increase. Controlling for gene-focused measures of male recombination did not affect the covariance between replication time and *K*_i_ (for the uncontrolled analysis Spearman's ρ = −0.081, *P* = 1.35×10^−6^; controlling for overall male recombination partial Spearman's ρ = −0.081, *P* = 0.001, Table 1). However, a slight increase in the strength of this relationship was indeed observed when controlling for smoothed measures of male recombination and was greatest for those that had shown the strongest positive covariance between recombination rate and replication time (controlling for mean-smoothed^2^ male recombination rates, partial Spearman's ρ = −0.09, p = 0.001, Table 1). Likewise, the lack of any relationship between *K*_i_ and all gene-focused measures of male-specific recombination was not affected by controls for replication time (*P* remained >0.05 for all, Table 1). However, a slight increase in the strength of the relationship between *K*_i_ and all smoothed measures of male recombination was observed (for the uncontrolled relationship between *K*_i_ and mean-smoothed^2^ male recombination, Spearman's ρ = 0.058, *P* = 0.0005; controlling for replication time, partial Spearman's ρ = 0.07, *P* = 0.001, Table 1).

Together, these results suggest that recombination might influence rates of evolution and interact with replication time by two separate sex-specific mechanisms, although the effects are modest. In estimating the impact of either timing of replication or recombination on the rate of neutral substitutions it is thus helpful, at the genic level, to perform a covariate controlled analysis, but as the correction is small, this isn't essential.

### Autosomal rates of evolution are better predicted by replication time than by recombination rates

The above analysis considered what happens when analysis is done at the genic level. But how can we understand between-autosome variation? For as yet unidentified reasons, more highly rearranged mouse autosomes have been found to have higher substitution rates (for the new dataset Pearson's *r* = 0.761, *P* = 0.0002; least squares linear regression *r* ^2^ = 0.579, *P* = 0.0002). As such, the extent of inter-autosomal rearrangement should be considered alongside any other parameters under investigation as predictors of between-autosomal variation in *K*_i_. To account for this a residuals test was therefore used whereby the residuals from the above regression were predicted by variation in the parameter of interest.

Previously it was shown that although replication time alone was unable to explain between-autosomal variation in rates of evolution, it was a significant predictor of this residual variation [8]. These findings were confirmed in the new dataset: although autosomal substitution rates do not covary with autosomal replication times (Pearson's *r* = −0.272, *P* = 0.26), residual variation in median *K*_i_ not explained by the rearrangement index could be predicted by differences in median timing of replication (*r* ^2^ = 0.237, *P* = 0.034), whereby earlier replicating autosomes have lower substitution rates than predicted by the rearrangement index and later replicating autosomes evolve faster than would be predicted by extent of rearrangement. When combined in a multiple least squares linear regression, rearrangement index and replication time could together explain 68% of inter-autosomal variation in *K*_i_ (*r* ^2^ = 0.679, *P* = 0.0001) and both parameters were significant predictors in this model (*P* = 4.89×10^−5^ for rearrangement index; *P* = 0.04 for replication time).

When autosomal recombination rates were subjected to a similar analysis, they too were found not to covary with autosomal rates of intronic evolution (for overall sex-averaged recombination rates Pearson's *r* = −0.182, *P* = 0.457). However, application of the same residuals test showed that unlike replication time, residual variation from the regression of *K*_i_ against rearrangement index could not be accounted for by autosomal recombination rates (for overall sex-averaged recombination rates *r* ^2^ = 0.018, *P* = 0.581). Further, the predictive power of the model to explain autosomal rates of evolution by the rearrangement index was only marginally increased by the inclusion of recombination rates (*r* ^2^ = 0.584, *P* = 0.00090) and recombination rate was not a significant predictor in the model (*P* = 0.00047 for rearrangement index; *P* = 0.673 for recombination rate). These findings were all robust to the use of alternative methods of assigning autosomal recombination rates and to the use of either male- or female-specific recombination rates (Tables S2 and S3).

That replication timing is a somewhat stronger covariate of *K*_i_ than recombination rate, particularly at the autosomal level, might in part be explained by the impact of extensive genomic rearrangements in the mouse lineage [44]. The high conservation of replication timing of homologous regions suggests that as sequences move around the genome, they tend to take their replication times with them [10], [45], [46]. In contrast, the relocation of rodent centromeres from a metacentric to a telocentric location has reduced the number of chromosome arms and, based on the requirement for at least one chiasma per arm, reduced the overall recombination rate of each autosome [47]. Further, recombination hotspots are known to be short lived [48], [49]. As such, while substitution rates and GC content are the product of processes occurring over long periods of time, the current replication time of a given sequences is more likely to reflect that to which it has been exposed to ancestrally than is the case for current recombination rates.

### GC content is better predicted by replication timing than by recombination rates

The current vogue suggests that the isochore structure of mammalian genomes is a result of recombination-associated biased gene conversion and that this process has a more profound effect in the male than in the female germline. However, early replicating sequences are known to be GC rich. Indeed more generally, a relationship between isochore boundaries and replication time boundaries is well described both on local and genomic scales [23], [24], [50], [51], [52]. Is then the local GC content better predicted by replication timing than recombination rate and how might we understand the result that male recombination, rather than female recombination appears to be relevant?

It is striking that timing of replication is a much stronger correlate of GC content (Spearman's ρ = 0.293, *P* = 5.34×10^−71^) than all measures of recombination rate (Spearman's ρ = 0.067, *P* = 6.44×10^−5^ for overall sex-averaged recombination, Table 1). Although the direction of the genic relationship was robust with highly recombining genes consistently having higher GC contents, the strength of the relationship was sensitive to gender: male-specific recombination rates being a stronger covariate of GC content than female-specific rates (Table 1). Methodology was also an important factor in determining the nature of the relationship. Gene-focused datasets were generally qualitatively similar. In contrast, the method of smoothing generated contrasting results: Use of medians to smooth both male and female recombination rates negated the significance of the relationship whilst for both genders the strongest correlate of GC content was mean-smoothed^2^ recombination rates (Table 1).

At the autosomal level, the contrast between replication timing and recombination rate as predictors was even more pronounced, with higher autosomal GC content correlating strongly with earlier autosomal replication (Pearson's *r* = 0.832, *P* = 9.83×10^−6^) but showing no covariance with autosomal recombination rates (Pearson's *r* = 0.376, *P* = 0.112 for overall sex-averaged recombination, Table S2).

In part, the relative weakness of recombination as a predictor may simply reflect less noise in the estimation of replication time, which has been shown to be conserved between species [10], [45], than in the effective ancestral recombination rate, recombination hotspots known to be fast evolving between even closely related species [48], [49]. Nonetheless, the above results suggests that the current focus on recombination associated biased gene conversion as the driver of isochores in mammals may be missing an important contribution from replication timing.

### The effect of female recombination on GC has been underestimated owing to interference from replication timing

The fact that highly recombining domains are GC rich has been taken as evidence that GC rich isochores are structured through gBGC (see [21] and references therein). Further, it has been suggested that this is a male-driven effect, with GC* (predicted equilibrium GC content) covarying more strongly with male than with female recombination rates [13], [27], [28], [29], [30], [31]. Indeed, recently, Clément and Arndt [32] noticed that GC content in rodents was well predicted by male specific recombination rates but not by female specific ones. They thus chose to ignore further consideration of female recombination as a potentially important cause of GC content. The findings presented here raise an interesting possibility: that the gender-specific nature of the impact of gBGC might be due to the differing relationships of recombination in each sex with replication timing. If we suppose there to be some force that promotes AT content in late replicating sequence, then if female recombination promotes AT→GC substitutions through biased gene conversion, this unknown force will oppose it. As a consequence, female recombination will leave a diminished footprint of AT→GC biased substitutions than that seen in male meiotic hotspots.

As expected by this model, significant relationships between GC content and female recombination were considerably increased when replication time was controlled for (for the uncontrolled analysis between GC and overall female recombination Spearman's ρ = 0.038, *P* = 0.025; controlling for replication time partial Spearman's ρ = 0.063, *P* = 0.001, Table 1). Indeed, the strength of the correlation, assayed using ρ^2^, between GC content and female recombination rates is more than doubled when controlling for replication timing (Table 1). By contrast, there is no perceptible change in the relationship between GC and replication time when controlling for any measure of female recombination (for the uncontrolled analysis Spearman's ρ = 0.293, *P* = 5.34×10^−71^; controlling for overall female recombination partial Spearman's ρ = 0.296, *P* = 0.001, Table 1).

For the influence of male recombination, if anything we expect the covariate uncontrolled analysis to over estimate as both early replication timing and higher recombination rates are associated with higher GC content. This is indeed what is observed and again the effect is greatest when the relationship between early replication time and high male recombination rate is strongest: For the uncontrolled analysis between GC and replication time, Spearman's ρ = 0.293, *P* = 5.34×10^−71^; controlling for overall male recombination, partial Spearman's ρ = 0.292, *P* = 0.001; controlling for mean-smoothed^2^ male recombination, partial Spearman's ρ = 0.278, *P* = 0.001 (Table 1). Similarly, for the uncontrolled analysis between GC and overall male recombination, Spearman's ρ = 0.078, *P* = 3.33×10^−6^; controlling for replication time, partial Spearman's ρ = 0.074, *P* = 0.001 and likewise for the uncontrolled analysis between GC and mean-smoothed^2^ male recombination, Spearman's ρ = 0.144, *P* = 6.96×10^−18^; controlling for replication time, partial Spearman's ρ = 0.109, *P* = 0.001 (Table 1). These effects appear to be relatively modest corrections, suggesting that the correlation between male recombination rates and local GC content is not grossly misleading.

## Discussion

This analysis was motivated by the hypothesis that, based on previously reported relationships with GC content, early replicating sequences would also be highly recombining. As rates of evolution have been found to be lower where replication is early, but elevated where recombination is higher, we therefore asked whether the two processes mask each other's impact on neutral substitution rates. While the use of sex-averaged recombination rates failed to support our initial assumption - that replication time and recombination rate covary - this masked a more important gender-specific complexity that has implications for our understanding of the causes of variation in substitution rate and GC content. These findings are robust to the range of alternative methodologies that we used to assign genic recombination rates. Unsurprisingly, we find that results are more pronounced when using mean-smoothed than noiser gene-focused datasets.

The idea that the influence of replication time and recombination on GC content may be in opposition is not new. Chen et al. [10] recently reported a greater increase in C∶G to A∶T substitutions compared to other substitution types as a function of time of replication through S-phase, possibly indicative of a decline in mis-match repair fidelity as replication proceeds. Although these authors note that the impact of replication timing might therefore counteract the increase in GC arising from gBGC, their use of sex-averaged recombination rates failed to identify that this process is particular to females. Our use of sex-specific data sheds new light on previous observations that gBGC appears to be a male driven phenomena, the impact of female-specific gBGC being possibly countered by later replication forcing higher AT content. This is important as the stronger covariance of GC* with cross-over rates in males than in females has been taken as evidence against a selectionist explanation for isochore evolution [21], [28].

As we have previously shown for rat [11], here we demonstrate a significant increase in intronic rates of evolution where mouse recombination rates are higher. In agreement with estimates in primates [10], in rodents this is at most of about the same magnitude as for replication time, if not weaker. Although we find that the magnitude of this relationship is overestimated in females and underestimated in males, the corrections are only modest. It is interesting to note that the overall relationship between *K*_i_ and crossover rates appears to be driven by recombination in females. This would suggest that our previous model of a male recombination-associated substitution effect to account for elevated and heterogeneous autosomal substitution rates [11] may require updating to include an additional or replacement female-specific recombination parameter.

The results here suggest that in order to fully understand the relationship between recombination rate and both GC content and substitution rates, we first need to understand how they relate to replication time. Understanding why the relationships differ with respect to gender may be key to this understanding. One possibility may be sexual dimorphism with respect to replication timing. The data we use here was derived from male ESC lines but whether these might differ from timings in females is not yet known. As highly expressed genes tend to replicate earlier in S-phase, one might suppose that differences in germline expression might give rise to such sex-specificity in replication time and that this in turn may explain our findings. With the possible antagonism between germline expression and recombination [53], [54], we can imagine a unified model in which differences in germline expression underpin both differences in replication timing and recombination. This we intend to leave for future analysis.

All the above results and discussion must by necessity come with the sizeable caveat that the correlations we describe do not necessarily imply causation. For example, the correlation between GC content and recombination rate might be because a) recombination alters GC content (e.g. via gBGC) [21] b) recombination is more common in GC rich domains [55] or c) GC content and recombination covary through a third hidden parameter (possibly gene expression). Further, although GC content and timing of replication are strongly correlated, it is not yet known which is causative of this relationship, nor why. More generally, the strong coupling between isochores and replication timing domains [23], [24], [50], [51], [52] remains both enigmatic and relatively under-explored. Indeed, recent attempts to explain mammalian isochore structure have focused on the role of recombination via the mechanism of GC-biased gene conversion [21]. Evidence for this comes, in part, from observations that recombination rate corresponds more strongly to GC* (predicted equilibrium GC content) than to current GC, suggesting that recombination is driving GC content [20], [28]. Experimental evidence [56] that gene conversion, at least in somatic cells, is biased in favour of GC residues over AT ones lends great credence to the model. In contrast, it is not clear whether the GC content determines replication time or *vice versa* (or neither) and there is evidence for both possibilities content (e.g. see [10], [40]). However, the findings presented here suggest that replication time appears to be as, if not more, important than recombination in relation to GC content.

If replication timing is important and causative of isochores then in principle this could be resolved via experimental assays. Consider for example the hypothesis that the high substitution rate in late replicating sequence is owing to error prone translesion synthesis [57]. If correct then this could in principle explain isochore evolution if translesion synthesis in mammals is biased towards the incorporation of A and T, thereby making late replicating sequence more AT rich. This prediction could in principle be examined in mammalian cell lines. Any model suggesting that replication timing causes isochores would also predict that GC rich sequence forced by deletion of early and strong replication origins to become late replicating should start to accumulate A and T.

## Supporting Information

Figure S1 **Covariance of unmasked and repeat-masked GC_i_.** Covariance of unmasked and repeat-masked intronic GC content. The dashed line represents *x* = *y*. The solid line is the orthogonal regression where Repeat-masked GC_i_ = −0.095+1.196949(Unmasked GC_i_).
(TIFF)

Figure S2 **Distribution of gene-focused recombination rates on chromosome 1.** Distribution of gene-focused female (red, upper plot) and male (blue, lower plot) recombination rates along chromosome 1. For both genders, the grey shaded plot is the recombination rate between every neighbouring pair of markers. Black dots in the centre of the plot represent genic positions. Lines represent overall (solid), weighted mean (dashed), interval mean (dotted) and interval median (dot/dash) recombination rates assigned to each gene.
(TIFF)

Figure S3 **Distribution of smoothed recombination rates on chromosome 1.** Distribution of smoothed female (red, upper plot) and male (blue, lower plot) recombination rates along chromosome 1. For both genders, the grey shaded plot is the recombination rate between every neighbouring pair of markers. Black dots in the centre of the plot represent genic positions. Dotted lines are mean-smoothed^2^ genic recombination rates. Solid lines are median-smoothed^2^ genic recombination rates.
(TIFF)

Table S1 **All genic Spearman's correlations using alternative datasets.** All genic Spearman's correlations between parameters X and Y calculated for this study, controlling for parameter Z in partial Spearman's correlations where appropriate. Ki = intronic substitution rate between mouse and rat; GC = repeat-masked intronic G+C content; RT_mean = genic mean replication time; RT_median = genic median replication time; RR = genic recombination rate curated using the alternative methodologies described in the column headings, for which SA = sex-averaged; M = male-specific; F = female-specific.
(XLS)

Table S2 **All autosomal Pearson's correlations using alternative datasets.** All autosomal Pearson's correlations between parameters X and Y calculated for this study, controlling for parameter Z in partial Pearson's correlations where appropriate. Ki = autosomal median intronic substitution rate between mouse and rat; GC = autosomal median repeat-masked intronic G+C content; RT_mean = autosomal median of mean replication times; RT_median = autosomal median of median replication times; RI = autosomal rearrangement index; RR = autosomal median recombination rates curated using the alternative methodologies described in the column headings, for which SA = sex-averaged; M = male-specific; F = female-specific.
(XLS)

Table S3 **All residuals tests for predictors of inter-autosomal variation in** ***K*** **_i_ using alternative datasets.** All results from residuals tests whereby inter-autosomal variation in *K*_i_ is predicted first by the Ki_predicitor. Residual variation from this regression is then predicted by Residual_predictor_1. Any residual variation in *K*_i_ from this second regression is then further predicted by Residual_predictor_2. Predictors are: GC = autosomal median repeat-masked intronic G+C content; RT_mean = autosomal median of mean replication times; RT_median = autosomal median of median replication times; RI = autosomal rearrangement index; RR = autosomal median recombination rates curated using alternative methodologies described in the column headings, for which SA = sex-averaged; M = male-specific; F = female-specific.
(XLS)
