# Supplementary figures and images for: Late Replicating Domains Are Highly Recombining in Females but Have Low Male Recombination Rates: Implications for Isochore Evolution
Source: PLoS One. 2011 Sep 20;6(9):e24480. doi: 10.1371/journal.pone.0024480 (PMC3176772; doi:10.1371/journal.pone.0024480)

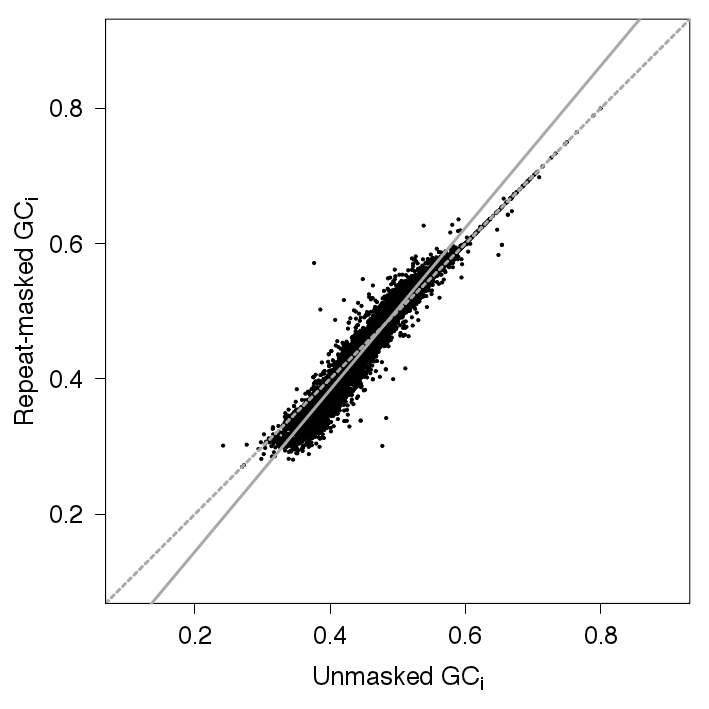

Supplement: Figure S1 — Covariance of unmasked and repeat-masked GCi. Covariance of unmasked and repeat-masked intronic GC content. The dashed line represents x = y. The solid line is the orthogonal regression where Repeat-masked GCi = −0.095+1.196949(Unmasked GCi). (TIFF) [file pone.0024480.s001.tiff]

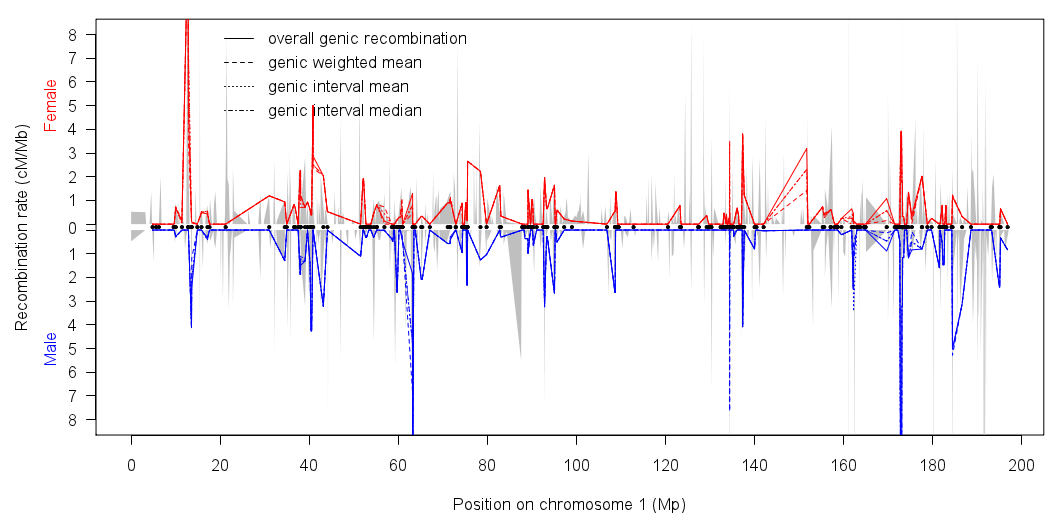

Supplement: Figure S2 — Distribution of gene-focused recombination rates on chromosome 1. Distribution of gene-focused female (red, upper plot) and male (blue, lower plot) recombination rates along chromosome 1. For both genders, the grey shaded plot is the recombination rate between every neighbouring pair of markers. Black dots in the centre of the plot represent genic positions. Lines represent overall (solid), weighted mean (dashed), interval mean (dotted) and interval median (dot/dash) recombination rates assigned to each gene. (TIFF) [file pone.0024480.s002.tiff]

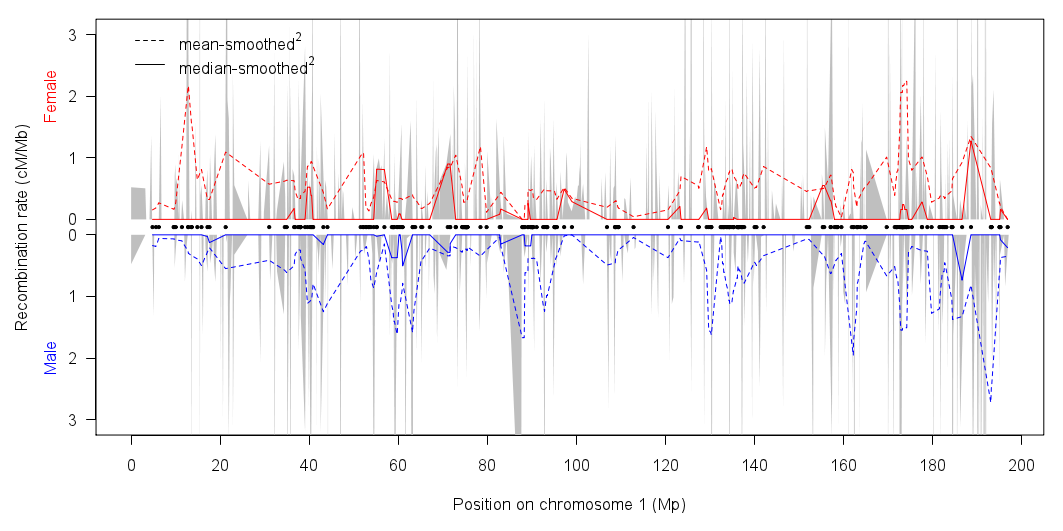

Supplement: Figure S3 — Distribution of smoothed recombination rates on chromosome 1. Distribution of smoothed female (red, upper plot) and male (blue, lower plot) recombination rates along chromosome 1. For both genders, the grey shaded plot is the recombination rate between every neighbouring pair of markers. Black dots in the centre of the plot represent genic positions. Dotted lines are mean-smoothed2 genic recombination rates. Solid lines are median-smoothed2 genic recombination rates. (TIFF) [file pone.0024480.s003.tiff]
